# The Arctic Human Health Initiative: a legacy of the International Polar Year 2007–2009

**DOI:** 10.3402/ijch.v72i0.21655

**Published:** 2013-08-05

**Authors:** Alan J. Parkinson

**Affiliations:** Arctic Investigations Program, Centres for Disease Control and Prevention, Anchorage, AK, USA

**Keywords:** International Polar Year, Arctic Health, research, education outreach communication, Arctic Council

## Abstract

**Background:**

The International Polar Year (IPY) 2007–2008 represented a unique opportunity to further stimulate cooperation and coordination on Arctic health research and increase the awareness and visibility of Arctic regions. The Arctic Human Health Initiative (AHHI) was a US-led Arctic Council IPY coordinating project that aimed to build and expand on existing International Union for Circumpolar Health (IUCH) and Arctic Council human health interests. The project aimed to link researchers with potential international collaborators and to serve as a focal point for human health research, education, outreach and communication activities during the IPY. The progress of projects conducted as part of this initiative up until the end of the Arctic Council Swedish chairmanship in May 2013 is summarized in this report.

**Design:**

The overall goals of the AHHI was to increase awareness and visibility of human health concerns of Arctic peoples, foster human health research, and promote health strategies that will improve health and well-being of all Arctic residents. Proposed activities to be recognized through the initiative included: expanding research networks that will enhance surveillance and monitoring of health issues of concern to Arctic peoples, and increase collaboration and coordination of human health research; fostering research that will examine the health impact of anthropogenic pollution, rapid modernization and economic development, climate variability, infectious and chronic diseases, intentional and unintentional injuries, promoting education, outreach and communication that will focus public and political attention on Arctic health issues, using a variety of publications, printed and electronic reports from scientific conferences, symposia and workshops targeting researchers, students, communities and policy makers; promoting the translation of research into health policy and community action including implementation of prevention strategies and health promotion; and promoting synergy and strategic direction of Arctic human health research and health promotion.

**Results:**

As of 31 March, 2009, the official end of the IPY, AHHI represented a total of 38 proposals, including 21 individual Expressions of Intent (EoI), and 9 full proposals (FP), submitted to the IPY Joint Committee for review and approval from lead investigators from the US, Canada, Greenland, Norway, Finland, Sweden and the Russian Federation. In addition, there were 10 National Initiatives (NI-projects undertaken during IPY beyond the IPY Joint Committee review process). Individual project details can be viewed at www.arctichealth.org. The AHHI currently monitors the progress of 28 individual active human health projects in the following thematic areas: health network expansion (5 projects), infectious disease research (7 projects), environmental health research (7 projects), behavioral and mental health research (4 projects), and outreach education and communication (5 projects).

**Conclusions:**

While some projects have been completed, others will continue well beyond the IPY. The IPY 2007–2008 represented a unique opportunity to further stimulate cooperation and coordination on Arctic health research and increase the awareness and visibility of Arctic regions.

The International Polar Year (IPY) was an intensive multidisciplinary program of collaborative international science, research, education and communication focusing on the Arctic and Antarctic regions. For logistical reasons, the IPY covered the 2-year period from March 2007 through March 2009 to allow for a full season of summer scientific activity in both the Arctic and Antarctic. The years 2007–2008 marked the 50th anniversary of the International Geophysical Year (IGY) and the third IPY. This event was designated the 4th IPY by the National Academy of Science, International Council of Science, the World Meteorological Organization, the Arctic Council and many other international organizations. This period of focused scientific activity promised to further our understanding of the physical and social process in polar regions, examine their globally connected role in the climate system and establish research infrastructure for the future, and serve to attract and develop a new generation of scientists and engineers with the versatility to tackle complex global issues (www.ipy.org) (1). In contrast to previous polar years, the IPY 2007–2009 had a much wider scientific scope including for the first time, fields of direct societal importance such as ecosystem and human health, and the development of indigenous societies and economics. The theme for the human dimension was established to “investigate the cultural, historical, and social processes that shape the sustainability of circumpolar human societies, and to identify their unique contributions to global cultural diversity and citizenship” (1).

## History of circumpolar health research

The scientific program of the IGY 1957–1958 did not have a human health component; however, it did provide the catalyst for the beginning of the “Circumpolar Health Movement” a collaborative international effort to focus on human health in the Arctic. In 1957, the Nordic Council appointed a committee for Arctic Medical Research that resulted in the publication of the Nordic Council for Arctic Medical Research Report. Also in 1958, the idea for an International Biological Program was conceived, and it was implemented in 1967 as a biological analogue for the IGY, which had served as a successful catalyst for Arctic and Antarctic research in the physical sciences (2).

Although human health is new to IPY activities, there is a well-established history of cooperation and collaboration in health research between polar nations. The first exploratory conference on Medicine and Public Health in the Arctic and Antarctic, sponsored by the World Health Organization (WHO), was held in Geneva from 28 August—to September 1962. It concluded that there was a need to stimulate high-latitude research especially on health problems (3). As a result of these combined events, the first international circumpolar health symposium was held in Fairbanks, Alaska, in 1967, and it was agreed to hold similar symposia every 3 years (4). Twenty years later, these meetings resulted in the formation of the International Union for Circumpolar Health (IUCH). The IUCH is a non-governmental organization comprising of an association of 5 circumpolar health organizations: the American Society for Circumpolar Health, the Canadian Society for Circumpolar Health, the Nordic Society for Arctic Medicine, the Siberian Branch of the Russian Academy of Medical Sciences and the Danish Greenlandic Society for Circumpolar Health. The IUCH promotes circumpolar collaboration and cooperation through the activities of its working groups in various fields of health and medicine (www.iuch.net). Outreach and communication are provided through the hosting of the triennial International Congress on Circumpolar Health (http://www.icch15.com).

The Arctic Council (www.arctic-council.org), established in 1996, is a Ministerial intergovernmental forum promoting cooperation, coordination and interaction between the 8 Arctic States (the US, Canada, Denmark/Greenland, Iceland, Norway, Sweden, Finland and the Russian Federation) including Arctic indigenous populations on common Arctic concerns such as sustainable development and environmental protection in the Arctic. Arctic Indigenous peoples are represented at the Arctic Council by Permanent Participant organizations Arctic Athabaskin Council, Aleut International Association, Gwitch'n Council International, Inuit Circumpolar Council, Russian Arctic Indigenous Peoples of the North and Saami Council. The scientific work of the Arctic Council is carried out in 6 working groups: The Arctic Contaminants Action Program (ACAP), the Arctic Monitoring and Assessment Program (AMAP), Conservation of Arctic Flora and Fauna (CAFF), Protection of the Marine Environment (PAME), Emergency Prevention Preparedness and Response (EPPR) and Sustainable Development Working Group (SDWG). The working groups conduct research and other activities in the areas of monitoring, assessing and preventing pollution in the Arctic; climate change; biodiversity conservation; emergency preparedness and response; sustainable development; and the monitoring and assessment of living conditions of Arctic residents including human health. The human health activities of the Arctic Council primarily reside in the AMAP and SDWG.

## IPY and the Arctic human health initiative

The Arctic Council recognized that IPY 2007–2008 represented a unique opportunity to further stimulate cooperation and coordination on Arctic health research and increase the awareness and visibility of Arctic regions. The Arctic Human Health Initiative (AHHI FP # 167) was a US-led Arctic Council IPY coordinating project that aimed to build and expand on existing Arctic Council and IUCH's human health research activities. The project aimed to link researchers with potential international collaborators and to serve as a focal point for human health research, education, outreach and communication activities during IPY. The overall goals of the AHHI was to increase awareness and visibility of human health concerns of Arctic peoples, foster human health research and promote health strategies that will improve health and well-being of all Arctic residents. Proposed activities to be recognized through the initiative included:

- Expanding research networks that will enhance surveillance and monitoring of health issues of concern to Arctic peoples, and increase collaboration and coordination of human health research;
- Fostering research that will examine the health impact of anthropogenic pollution, rapid modernization and economic development, climate variability, infectious and chronic diseases, intentional and unintentional injuries;
- Promoting education, outreach and communication that will focus public and political attention on Arctic health issues, using a variety of publications, printed and electronic reports from scientific conferences, symposia and workshops targeting researchers, students, communities and policy makers;
- Promoting the translation of research into health policy and community action including implementation of prevention strategies and health promotion; and
- Promoting synergy and strategic direction of Arctic human health research and health promotion.

As of 31 March 2009, the official end of the IPY, AHHI represented a total of 38 proposals, including 21 individual Expressions of Intent (EoI), and 9 full proposals (FP), submitted to the IPY Joint Committee for review and approval from lead investigators from the US, Canada, Greenland, Norway Finland, Sweden and the Russian Federation. In addition, there were 10 National Initiatives (NI-projects undertaken during IPY beyond the IPY Joint Committee review process). Individual project details can be viewed at www.arctichealth.org.

The AHHI currently monitors the progress of 28 individual active human health projects in the following thematic areas: health network expansion (5 projects), infectious disease research (7 projects); environmental health research (7 projects); behavioral and mental health research (4 projects); and outreach education and communication (5 projects). While some projects have been completed, others will continue well beyond the IPY. The progress of these projects is summarized in this report (Table I).

**Table I:** Active Arctic Human Health Initiative (AHHI) Proposals as of March 31, 2009

Project Title	Lead Country(s)	EoI/FP#
**Expansion of Networks**		
International Circumpolar Surveillance	USA	1150
International Network for Circumpolar Health Researchers http://www.inchr.com/	Canada	516
Arctic Health Research Network http://www.arctichealth.ca/	Canada	**449**
Survey of Living Conditions in the Arctic: Remote Access	Denmark	**386**
Arctic Community-Based Environmental Monitoring, Observation and Information Stations Phase 1: Bering Sea Sub-network	USA	922
**Research**		
The Inuit Diet and Health Study: Inuit Health in Transition	Canada	**253**
Integrated Research on Arctic Marine Fat and Lipids		NI1
Inuit Health Survey: Inuit Health in Transition and Resiliency (http://www.inuithealthsurvey.ca/?nav=home)		NI2
Genetics and Environmental Risk Factors for Complex Diseases: A study of the Saami population	Sweden	1274
Center for Alaska Native Health Research	USA	NI3
Does Exposure to Persistent Organic Pollutants (POPs) increase the risk of breast cancer?	Denmark	1257
An Epidemiological Study of the Cumulative Health Effects of POPs and Mercury in Subsistence Dependent	USA	NI4
Rural Alaska Natives.		
The burden of Infectious Diseases in Greenland-means of evaluation and reduction	Denmark	1107
Hepatitis B in aboriginal Populations in the Arctic: Alaska Natives, Canadian Inuit, First Nations Peoples, Greenland Inuit and Russian Native Populations.	USA	1109
Addressing Viral Hepatitis in the Canadian North	Canada	NI5
Sexual Health and Sexually Transmitted Infections in Northern Frontier Populations.	Canada	1147
Engaging Communities in the Monitoring of Zoonoses, Country Food Safety and Wildlife Health	Canada	**186**
Evaluation of the impact of an immunization program combining pneumococcal conjugated vaccine and inactivated influenza vaccine in Nunavik children, Province of Quebec, Canada	Canada	1119
Prevalence of Human Papillomavirus Infection and Cervical Dysplasia in the North West Territories	Canada	1121
*Health and social condition of adoptees in Greenland - a comparative register* and population based field study.	Denmark	1201
Creation of an “adoptees-database”		
Healthy Lifestyle Projects	USA	1271
*Negotiating Pathways to Adulthood: Social Change and Indigenous Culture in 4 Circumpolar Communities*	USA	1266
Mental and Behavioral Health Issues in the U.S. Arctic	USA	NI6
** Outreach, Education, Communication:**		
*The Circumpolar Health and Wellbeing: Research program for Circumpolar Health and Wellbeing, Graduate School of Circumpolar Wellbeing, Health and Adaptation, and International Joint Master's Program in Circumpolar Health and Wellbeing*	Finland	1045
*Scientific and professional supplements on human health in polar regions-the International Journal of Circumpolar Health*	Finland	1046
Development of a Women's Health and Well-Being Track at the 14th International Congress on Circumpolar Health in Yellowknife, NWT July 2009	USA	1223
Telemedicine Cooperation Project	USA	1270
Arctic Monitoring and Assessment Program Human Health Assessment Group Conference.	Canada	145
Climate Change and Impacts on *Human Health in the Arctic: An International Workshop on Emerging Threats and Response of Arctic Communities to Climate Change*	USA	NI7
** Canadian training, communications and outreach projects**		
The Inuit Cohort: A Community of Research Practice Across Canada http://www.ciet.org/en/documents/projects_cycles/2007102165919.asp		NI8
Healthy Foods North NWT http://www.hlthss.gov.nt.ca/sites/healthy_foods_north/default.htm		NI9
Pan-Arctic Interactive Communications Health Project http://www.naho.ca/inuit/wellnessTV/index.php		NII0

# The table lists proposals by lead country submitted to the Joint Committee as Expressions of Interest (EoI), or Full Proposal (FP-in bold). Projects undertaken during IPY beyond the IPY Joint Committee review process are listed as National Initiatives (NI).

## Expansion of research networks

The establishment of well-coordinated and Sustained Arctic Observing Networks (SAON) was a major objective of the IPY (www.arcticobserving.org). The goal was to develop long-term Arctic-wide observing activities that provide free, open and timely access to high-quality data for both the scientific and societal communities. In 2006, the Arctic Council Ministers requested that the AMAP together with other Arctic Council working groups and external partners create a coordinated Arctic Observing System to monitor Arctic Change. One of the priorities of the SAON process is to identify existing observing networks and opportunities for improving access and data sharing.

Several circumpolar human health monitoring networks already exist and could form the basis for the establishment of a SAON for human health. These currently include the AMAP Human Health Assessment Program, the International Circumpolar Surveillance (ICS) of Infectious Diseases and the Circumpolar Health Observatory (http://circhob.circumpolarhealth.org). Together these networks could provide:

- An international circumpolar collaborative health information system;
- Systematic standardized, consistent methods in data collection, analysis and reporting;
- Ability to monitor trends and patterns in health status, health determinants and health care;
- Quantitative evidence for planning and evaluation of health programs and services; and
- A system that is population based and aggregated by administrative regions in all circumpolar countries.

Existing networks that could provide the basis for such an observing system include:

### International circumpolar surveillance

Established in 1999, the ICS system is an integrated population-based infectious disease surveillance network system, linking hospital and public health laboratories in the Arctic Circumpolar countries (USA/Alaska, Canada, Iceland, Greenland, Norway and Finland) (5,6). Accomplishments during IPY included: an expansion of surveillance to include tuberculosis; an effort to include northern regions of the Russian Federation in this system; and the establishment circumpolar working groups to focus on research aspects of viral hepatitis (EoI # 1109), diseases caused by *Helicobacter pylori*, and sexually transmitted infections (STIs) (EoI #1150).

The purpose of the ICS system for infectious diseases is to establish a surveillance network of hospital and public health laboratories throughout the Arctic (7). The network allows the collection and sharing of uniform laboratory and epidemiologic data between Arctic countries that defines the prevalence of infectious diseases of concern to Arctic residents and assists in the formulation of prevention and control strategies (8–10). While currently focused on prevention and control of infectious disease, the system could be adapted to monitor other human health issues of concern in Arctic countries and serves as a model for a Sustainable Arctic Observing Network for human health.

### Arctic Monitoring and Assessment Program: Human Health Assessment Group

As part of the IPY a joint Arctic Monitoring and Assessment Program (AMAP) and Northern Contaminants Program (NCP) symposium was held in Iqaluit, Nunavut, Canada, from 10–12 June 2009 (FP # 145). The AMAP has been coordinating circumpolar monitoring and assessment of atmospheric pathways, biota impacts, food chain dynamics and human health issues for environmental contaminants since 1991 (http://www.amap.no/). The contaminants have included persistent organic pollutants (POPs – both historic and emerging compounds), metals, and radionuclides of concern in the circumpolar world. The AMAP–Arctic Human Health Assessment Group (AHHAG) has members in all 8 circumpolar countries and has completed 3 assessments on the human health impacts of arctic environmental contaminants (11–13). These assessments include human monitoring data, dietary studies, health effects studies and risk management strategies to mitigate the effects of contaminants. The AHHAG has effectively functioned as an Arctic Observing Network for environmental contaminants in the circumpolar north and could work with the other human health observation networks to give an integrated picture of circumpolar human health.

### International Network for Circumpolar Health Research

The IPY saw the establishment of the International Network for Circumpolar Research (INCHR) (EoI #516). This is a voluntary network of individual researchers, research trainees, and supporters of research based in academic research centres, Indigenous people's organizations, regional health authorities, scientific/professional associations and government agencies, who share the goal of improving the health of the residents of the circumpolar regions through international cooperation in scientific research (www.inchr.com). The goals of INCHR are to:

- Conduct, sponsor and promote research programs and projects investigating the patterns, determinants and impact of health conditions among circumpolar peoples and the strategies for improving their health;
- Support research training at all levels and increase capacity for circumpolar health research in communities, service delivery agencies and higher educational institutions;
- Facilitate exchange, communication and dissemination of research data; and
- Strengthen the health information system in the circumpolar region.

In 2012, INCHR was merged with the International Association of Circumpolar Health Publishers to form the Circumpolar Health Research Network or CircHNet (http://circhnet.org) and became the publisher of the International Journal of Circumpolar Health (www.circumpolarhealthjournal.net). CircHNet will continue the work of INCHR in organizing annual scientific conferences, summer schools in health research and support for international exchanges of scientists and trainees.

### Arctic Health Research Network

The Arctic Health Research Network (AHRN) was launched as a Canadian contribution to IPY 2007–2008 (EoI # 449; http://www.arctichealth.ca/aboutahrn.html) and was supported by a tri-territorial health fund. The Arctic Health Research Network is a health research network based in the 3 northern territories and a provincial region of Canada. The network aimed to build capacity for northern based health and wellness research though the development of 4 sites in Yukon, Northwest Territories, Nunavut and Labrador. Each was developed under independent boards and is registered under the territorial societies Acts. The initiative supported the development of 3 institutes based in the 3 northern territories and a provincial region of Canada and has 4 sites in Yukon, Northwest Territories, Nunavut and Labrador. The institutes developed included the Institute for Circumpolar Health Research (www.ichr.ca), the Arctic Institute of Community-Based Research (AICBR) (www.aicbr.ca) and the Qaujigiartiit Health Research Centre (AHRN-NU) (http://www.qhrc.ca). Each organization aims to respond to, and provide leadership for, northern regional health and wellness research needs.

### Survey of Living Conditions in the Arctic-Remote Access

The Survey of Living Conditions in the Arctic (SLiCA, FP # 386) is an interdisciplinary and international research project, which was founded in 1998 (14,15). The project is developed in partnership with the local indigenous peoples organizations. SLiCA has accomplished data collection in Canada, Alaska, Chukotka (Russia), Greenland and Sweden (16), and by the end of 2008, interviews among the Sámi in Norway and the Kola Peninsula were concluded. The data material consists of approximately 8,000 personal interviews. During IPY, SLiCA intended to expand the understanding of Arctic change by extending the concepts of remote access analysis to the SLiCA international database (17), allowing other researchers to remotely conduct analysis without access to raw data. All interview data (except the Canadian SLiCA data) have been included in an SPSS database and almost 600 tables including survey results based on the interviewing among the Inuit (www.arcticlivingconditions.org). The first phase of this project developed a standardized research design for the measurement of living conditions and well-being among the Inuit, Saami and indigenous peoples of Chukotka (18). The survey was completed in 2006.

### The Bering Sea Sub Network: International Community-Based Environmental Observation Alliance for the Arctic Observing Network Survey of Living Conditions in the Arctic-Remote Access

The Bering Sea Sub Network: International Community-Based Environmental Observation Alliance for the Arctic Observing Network, known as BSSN (FP #922), was a 2008–09 IPY project implemented by the Aleut International Association in collaboration with the University of Alaska, United Nations Environment Programme–Global Resource Databank Arendal and the Alaska Native Science Commission under the auspices of the CAFF working group of the Arctic Council. BSSN is funded by the United States National Science Foundation. The project began as a pilot in 2007 and received an award for a 5-year continuation for Phase II in 2009.

BSSN is a network of coastal communities. It began from 6 villages representing 6 indigenous cultures: 3 in the Russian Federation (Kanchalan–Chukchi population; Tymlat–Koryak population and Nikolskoye–Western Aleut/Unangas population) and 3 in the United Stated (Gambell–St Lawrence Island Yupik; Togiak–Central Yu'pik; and Sand Point–Eastern Aleut/Unangan). During Phase II Savoonga (St Lawrence Island Yupik) and Saint George (Eastern Aleut/Unangan) were added to the network. This project creates a structured framework that provides the means for the systematic collection of information about the environment and lays a foundation for future community-based research. The network also provides for the efficient management of data gathered from community-based environmental observations.

The overall goal of the BSSN is to improve knowledge of the environmental changes that are of significance to understanding pan-arctic processes, and to enable scientists, arctic communities, and governments to predict, plan, and respond to these changes.

BSSN's objective was to develop a framework to enable residents in remote Arctic communities to systematically document physical and social changes occurring in their region. This may enhance community resilience under conditions of rapid environmental and social change.

BSSN has emerged as an observing network that connects people bound by a common geographic area who share similar traditions, values and ideals (19–21).

## Research

IPY human health research focused on some of the issues of most concern to Arctic residents. These include: the health impacts of environmental contaminants, climate change, rapidly changing social and economic parameters within communities, the changing patterns of infectious and chronic diseases, and the continuing health disparities that exist between indigenous and non-indigenous segments of the Arctic populations. While other issues of importance such as injuries and maternal child health were not directly addressed by specific proposals during the IPY and are thus not covered in this report, they do appear as research outputs in broader spectrum outreach, education and communication activities (22).

The intensity of research activities and networks during IPY has served as a catalyst to integrate programs, and to promote the concept of communities and researchers working collaboratively. It was hoped research informed by community perspectives would enhance the eventual translation of research into policies and programs that will improve circumpolar health.

### Environmental contaminants

While socio-economic conditions and lifestyle choices are major determinants of health, contaminants may also have a contributing effect. Toxicological studies show that contaminant levels found in some parts of the Arctic have the potential for adverse health effects in people who rely on a traditional diet for their subsistence. These include the indigenous peoples of the Arctic. Epidemiological studies looking at Arctic residents directly provide evidence for subtle immunological cardiovascular and reproductive effects due to contaminants in some Arctic Indigenous populations (13). If climate change is associated with rising salmon and human levels of POPs and mercury the study would provide data to further support reduction of POPs and mercury production and release, and efforts to reduce global warming.

A US-led study, initiated by investigators at the Alaska Native Tribal Health Consortium, examined the cumulative health effects of POPs and Hg in subsistence dependent rural Alaska Natives (NI4). The objectives of the study are to determine time trends in tissues levels of POPs, mercury and omega 3 fatty acids in salmon in the Yukon and Kuskokwim rivers and in a cohort of 200 pregnant Alaska Native Yupik women and their infants. Prior work by this group started in 1998 and contributed data to the AMAP Human Health Assessments in 2002 and 2009 (12,13). This early study in a cohort of 354 Alaska Native Yupik women showed that in general, legacy organic pollutants and mercury levels in these women are quite similar to maternal blood levels from Scandinavian, Icelandic and Inuit women from the western Canadian Arctic. Levels are generally lower than Inuit women from the eastern Canadian Arctic, Greenland, and the Russian Far East. The exceptions are levels of brominated flame retardants (BFRs) and levels of polyfluorinated compounds (PFCs) which are much higher in Alaska Native Yupik women than any other Arctic maternal AMAP populations. Preliminary conclusions thus far show that in this population, the close association of mercury, omega-3 fatty acids, organochlorines and PFCs suggest that the northern marine subsistence diet is the source of these contaminants and micronutrients. Analysis of health outcomes of mothers and infants, along with possible associations with analytes have yet to be carried out.

Another study led by researchers at the Center for Arctic Environmental Medicine, School of Public Health, University of Aarhus, Denmark, examined the risk of the development of breast cancer in Greenlandic Inuit women following exposure to POPs (EoI #1257). Blood levels of POPs in women with breast cancer were compared to controls with respect to age and lifestyle. The bio-effects of POP levels on hormone receptor function were examined (23). The incidence of breast cancer has been traditionally low among the Inuit, but a considerable increase has been observed since the 1970s with rates now approaching respective national populations (22). Previous data in Greenlandic Inuit women suggest that exposure to POPs might contribute to the risk of breast cancer. Rat studies showed that PFCs cause significantly increase in mammary fibroadenomas. This study aimed at evaluating the association between serum levels of POPs/PFCs in Greenlandic Inuit breast cancer cases and their controls, and whether the combined POP related effect on nuclear hormone receptors affect breast cancer risk. Results showed for the first time a significant association between serum PFC levels and the risk of breast cancer (24–26). Further investigations are needed to document the study conclusions.

### Infectious diseases

A continuing major health disparity is the increased morbidity and mortality due to infectious diseases seen among indigenous populations when compared to the non-indigenous populations of the Arctic. These disparities can be resolved with greater understanding of their causes through research and focused efforts at treatment and prevention.

Hepatitis B infection occurs at high and endemic rates in Arctic populations. For example, in the past, research had shown that 3–5% of individuals residing in the Canadian North, 5–14% of Inuit in Greenland and 3–10% of Alaska Native people in Western Alaska were infected with hepatitis B virus (HBV) and likely that, if left untreated, 10–25% could develop liver cancer or die of cirrhosis. Researchers from the US, Canada, Greenland, Denmark and the Russian Federation have formed a Circumpolar Viral Hepatitis Working Group and are conducting studies to determine the epidemiology of chronic HBV in indigenous populations (EoI #1109). The study monitors patients to determine disease progression; demographic characteristics associated with disease outcome; environmental factors associated with disease outcome including contaminants in the environment and subsistence foods; cofactors such as alcohol intake, obesity and metabolic syndrome; viral characteristics such as genotype, viral load and mutations that could affect disease outcome. This study allows the identification of barriers to vaccination, the development of registries for research and clinical management and the development of criteria to identify potential treatment candidates, monitoring of treatment outcome and the examination of the role of factors such as demographics, viral genotype and environmental factors in treatment outcome. Already this research group has identified a new HBV sub-genotype (B6), which is only found in indigenous populations of Alaska, Canada and Greenland (27). The group also assisted Greenland in the investigation of an outbreak of hepatitis D super infection in adolescents with chronic HBV in a community in Greenland (28). In addition, this working group has been instrumental in encouraging the Greenland government to adopt universal childhood hepatitis B vaccination in Greenland (29). A Canadian-led IPY study (NI5) examined the genetic diversity of HBV genotypes B6, D and F among circumpolar indigenous individuals and found mutation rates significantly higher in the form of (B6) present in the Canadian Inuit (30). The Canadian study also examined the prevalence and long term out-come of occult hepatitis B (where only viral DNA and no serological markers of infection are detectable in blood). They studied 3 northern Canadian populations and found that occult hepatitis B is less common than hepatitis B and was not associated with any long term adverse clinical outcome (31).

Similarly reported rates of STIs are disparately high among indigenous populations of the Arctic (32). Research in Canada, the US, and Greenland (EoI #1147) aimed at building capacity to examine individual, social and environmental factors that influence perceptions of sexual health and STIs is being conducted by researchers and communities using participatory methods (33,34). The aims include a description of the basic epidemiology of sexual health and STIs and to identify communities at risk and targets for capacity building and interventions. Preliminary results indicate that *Mycoplasma genitalium* is as prevalent as *Chlamydia trachomatis* in Greenland and that social and cultural norm around sexual health communication, trust, drinking and sex appear to influence individual sexual behaviors and risk for STIs (35). Based on this research, the National Science Foundation has granted US, Canadian, Greenlandic and Danish researchers new funds to explore community based participatory methods in Greenland and develop a social intervention focusing on sexual health communication with families and relationships.

Canadian researchers are examining the potential for incorporating Human Papillomavirus (HPV) DNA testing into the present screening program (EoI #1121). This project examined HPV infection and cervical dysplasia (precancerous cells) in women of the Northwest Territories, Yukon, Nunavut and Labrador to determine general prevalence rates, types of HPV and risks associated with the development of HPV. The aim is to provide scientific evidence for policymakers and local public health workers to assist in the planning and implementation of cancer control programs. Results from 14,598 bio-samples showed an overall HPV prevalence 25.2%, of which 78.6% with high risk types and 32.5% with multitypes infection. The HPV prevalence was approximately 40% higher among the aboriginal than the non-aboriginal population, overall and in most of the age-groups. The prevalence of HPV infection was elevated in the young aboriginal population in the NWT (36–38). HPV infection attributes to more than 80% of abnormal cervical cytology cases. An effective vaccine program may reduce the cervical abnormality to lower than half of its current level.

With their strong hunting traditions and subsistence based on wild game, Arctic indigenous peoples are at increased risk for zoonoses and parasitic infections acquired from infected meat. Zoonoses refer to a group of diseases caused by organisms that are usually present in animals but are transmitted to and cause disease in humans. As temperatures warm and habitats change, some parasites could move northward with the migration of their wildlife hosts, and others will increase their density due to optimal temperatures for replication. These factors, together with other environmental changes (water availability, ice and snow cover, ocean currents, extreme weather events, forest fires), will favour a shift in the distribution of hosts and zoonotic diseases threats to the safety of traditional subsistence foods (39,40). Food-borne parasites such as *Trichinella sp*. and *Toxoplasma gondii* are significant Arctic zoonoses endemic in some regions and are directly related to consumption of country food (41,42). Others such as *Anisakidae* nematodes and the bacteria *Salmonella sp*. and *Escherichia coli* 0157:H7 can become a zoonotic issue with warmer weather. A study in Canada has resulted in the development of simplified pre-screening diagnostic tests for *Salmonella sp*. and *Escherichia coli* 0157:H7 (43) and into the development of qPCR techniques and multispecies ELISA for *Toxoplasma gondii* detection (EoI# 186). The study provided equipment and training for northerners to collect samples and the evaluation of some of these tests in 3 northern communities. Results show that *Trichinella* infection is present in northern carnivore mammals, except for seals and beluga. The 2 species present, *Trichinella nativa* and T6, are freeze-resistant. Collection and storage of blood using a filter paper technique is useful for *Toxoplasma* detection but needs to be validated with other diseases (44). *Anisakidae* nematodes are present in marine fish and mammals traditionally eaten by eastern Canadian Inuit. Data of each disease studied in Canadian wildlife will be included into the Canadian Cooperative Wildlife Health Centre database linked to the IPY database centre.

The ICS project has shown that *Streptococcus pneumoniae* is one of the leading causes of pneumonia, meningitis, bacteremia, septic shock and otitis media in Arctic indigenous populations, particularly among children and the elderly (8). For example, the incidence rates of invasive pneumococcal disease in Inuit are approximately 4 times that of non-Inuit. A Canadian study is retrospectively analyzing immunization and laboratory records of persons living in Nunavik to describe the epidemiology of invasive pneumococcal disease in relation to vaccine use during the period 1997–2010 (EoI # 1119). The implementation of vaccine programs in this region in 2002 controlled an outbreak of invasive pneumococcal disease in young adults caused by pneumococcal serotype 1. Use of the 7-valent conjugate vaccine in children markedly reduced the rate of disease caused by these 7 most common serotypes in children, but did not prevent disease caused by non-vaccine types (45). The impact of a 13-valent vaccine is being evaluated in Nunavik. An evaluation of the impact of the 7-valent vaccine on hearing loss in children showed that the vaccine had no significant impact on reducing major audiology disorders (46).

The IPY provided the opportunity to strengthen surveillance and research on infectious diseases in Greenland (EoI #1107). This project, a cooperation between Greenland and Denmark, addressed the burden of infectious diseases in Greenland by establishing research programs to evaluate long-term consequences of certain infectious diseases, to evaluate the use of routine surveillance data, to initiate intervention trials in order to prevent infectious diseases, to seek implementation of results in the Greenland health system and to establish cooperation with public health and research organizations in other countries. Specific studies under this project included a validation of the Greenlandic inpatient register, the initiation of tuberculosis studies (47–52), an evaluation of the distribution of bacterial pathogens causing invasive disease (8,53,54), a study of the long-term consequences of hepatitis B (27–30,55), a study of the association between Epstein Barr virus and various cancers (56,57), a study of HIV drug resistance (58,59), a study of HIV and living conditions (60), a study on gene mutations and hearing (61), longitudinal studies on chronic otitis media (62,63), a study of the first case of Q fever endocarditis in Arctic Areas (64) and a study of the aetiology of viral respiratory pathogens among Greenlandic children.

In collaboration with Canadian researchers, a nationwide study of viral pathogens in children hospitalized with lower respiratory tract infections in Greenland is ongoing. With researchers in Canada and the USA, the network organization is involved in studies of epidemiological, microbiological and social aspects of STIs (EoI # 1109) (33–35).

These activities have resulted in the creation in 2012 of a network of circumpolar infectious disease researchers by the Greenlandic, Danish and US governments as part of a capacity building bilateral project to promote continued collaboration, sharing of results and best practices to investigate impact of climate change on infectious diseases, and builds on existing infectious disease collaborations. A purpose is to encourage early career researchers and indigenous peoples to participate in an international network of researchers.

### Life-style, diet and nutrition

Considerable life-style changes have occurred over the past decades among the indigenous peoples in the circumpolar region. Parallel to this has been a change in disease patterns, with an increase, for example, in cardiovascular diseases, obesity and diabetes. Among the main causes are alterations to the diet and decreased levels of physical activity as the population changes from their traditional hunting and fishing economy to more Westernized living conditions. Several large IPY activities were initiated to address some of these issues.

A large international study entitled “The Inuit Health in Transition (EoI #760; NI1, NI2) and Inuit Diet and Health Study (FP #253)” was proposed to cover a cohort of over 7,000 Inuit adults in Alaska, Canada, and Greenland during IPY.

The Government of Canada Federal Program for IPY funded a major component of this international study during 2007–08 in the Inuvialuit Settlement Region (ISR) of the Northwest Territories, Nunavut and the Nunatsiavut region of Labrador. Known as the IPY-Inuit Health Survey, and utilizing the Canadian coast guard ship Amundsen, which was equipped with research and laboratory facilities, 33 coastal communities were visited and 3 inland communities were visited by separate survey teams (65). A total of 1,901 households participated (68%), with a total of 2,595 participants aged 18 years or older. The cross-sectional adult survey provides baseline data concerning the risk factors for cardiovascular disease and type 2 diabetes mellitus in the Inuit undergoing acculturation as well as evaluates social support and other determinants of resiliency and self-reported health. Matching funding from the Northern Contaminant Program in Canada also supported the research on dietary contaminants exposure associated with country food consumption.

The most pressing health concerns for Inuit adults were food insecurity (66,67), overweight and obesity and the emergence of obesity-related chronic diseases (68), iron deficiency in women of reproductive age (69,70), vitamin D deficiency (71) and the high obesogenic potential of high sugar-drink consumption (72,73). These health priorities are interlinked in the context of economic disadvantages and high market food costs in the Arctic. Most Inuit Health Survey participants had blood contaminant concentrations below guidelines set by Health Canada even though metal and POPs body burdens commonly exceed exposures observed in the general population of Canada (74). Results on contaminant–nutrient interactions showed a strong correlation between mercury (Hg) and nutrients (selenium and n-3 fatty acids), suggesting that efforts to decrease Hg exposure must emphasize the overall healthfulness of traditional foods and be designed to prevent concomitant harm to the nutrient intakes of Inuit (75,76).

In addition, 388 children aged 3–5 years from 16 Nunavut communities took part in the Nunavut Inuit child health survey. The goal of the child health survey was to evaluate nutritional status, breastfeeding and complementary feeding practices, food security, access to country food, respiratory tract and ear infections, and diet quality. Results from the child survey indicated that nearly 70% of Inuit preschoolers resided in households rated as food insecure [69.6%; 95% confidence interval (CI) 64.7–74.6%]. Overall, 31.0% of children were moderately food insecure, and 25.1% were severely food insecure, with a weighted prevalence of child food insecurity of 56.1% (95% CI 51.0–61.3%) (77). Furthermore the overall prevalence of overweight was 50.8% (78) and vitamin D deficiency and insufficiency were highly prevalent among Inuit preschoolers living in Nunavut (79). Nearly 25% of children had hair Hg concentrations equal to or higher than 2 µg/g (WHO reference level). There was a significant correlation between mercury levels in children's hair and that of the adults in the same household. For children, beluga muktuk, narwhal muktuk, ringed seal liver, fish, caribou meat and ringed seal meat were the major dietary sources of mercury. Both local and international policies are needed to lower the intake of dietary Hg exposure among Inuit children in the circumpolar north.

A Swedish IPY project evaluates a northern Swedish population with known demographic and environmental exposures to identify genetic and environmental factors that contribute to health status (EoI #1274). In this study, cross-population comparisons are used to study genetic and environmental risk factors among populations with widely differing origins and environments. The study measures a broad spectrum of environmental (e.g. diet, physical activity and daylight exposure) and genetic (e.g. single-nucleotide polymorphisms) factors with potential relevance for health risk. A comprehensive set of health indicators and diagnoses of cardiovascular, orthopaedic and metabolic diseases has been collected. The laboratory analysis of blood lipids comprising several hundreds of lipid species will give unique insights into the human metabolism under extreme living conditions. Studies of rural populations can make substantial contributions to basic research to understand environmental and genetic determinants of disease. The European Special Population Network (EUROSPAN) provides a platform combining studies of rural populations from different parts of Europe to leverage these for collaboration with large international consortia (80–82).

In the US the Center for Alaska Native Health Research (CANHR) at the University of Alaska Fairbanks used the IPY momentum to build a collaborative research presence in Alaska Native communities (NI4). Research focused on prevention and reduction of health disparities by seeking new knowledge through basic and applied research that can ultimately be applied to understand, prevent and reduce health disparities in indigenous communities (http://canhr.uaf.edu/) (83). The centre studies behavioral, dietary and genetic factors related to obesity diabetes and cardiovascular disease risk in Alaska Natives of south-western Alaska. CANHR includes studies related to substance abuse and suicide prevention, the development of novel dietary biomarkers, contaminants and the safety of subsistence foods, stress, gene by environment interactions and nutrition research. All CANHR studies employ community based participatory research approaches.

### Behavioral and mental health

Behavioral and mental health disorders are common worldwide and circumpolar regions are not exempt from this burden. Contemporary dynamics of rapid social change have dramatically affected the political, cultural and economic systems of circumpolar indigenous people. Alcohol abuse and suicide have been highlighted as significant issues in northern regions (84,85). During IPY, there were a number of research projects which explored behavioral and mental health disorders and the relationships between outcomes and environmental factors, including social determinants.

The Inuit Health Survey collected information on mental and community wellness. Findings will provide information on the burden of mental illness, and also evaluated social support and other determinants of resiliency and self-reported health (86). In Nunavik, a cohort study was carried out which focused on exposure to environmental contaminants and child behavior. The study also explored the impact of lifestyle factors, such as smoking, alcohol and drug abuse during pregnancy, on multiple domains of child development and behavior (87).

Two CANHR-affiliated studies focus on behavioral health research. This US-led study examined social change and indigenous culture in 5 circumpolar communities by exploring responses to rapid social transition through the life experiences of circumpolar youth in order to identify resilience processes that might guide prevention, treatment and policy (EoI # 1266). This study completed over 100 youth life history interviews from Alaska Inupiat, Alaska Yup'ik, Canadian Inuit, Norwegian Saami and Siberian Eveny communities. The project team identified shared and stressors and patterns of resilience in the transition to adulthood across these different circumpolar settings along with innovative approaches in youth-driven participatory research (88,89).

Elluam Tungiinun – “Toward wellness” – is a culturally based preventive intervention to reduce suicide risk and co-morbid underage drinking among Alaska Native Yup'ik Eskimo youth. This study represents the next stage in a 15-year community-based participatory research process with Alaska Native people (90–92). The goal is to identify protective factors from alcohol abuse and suicide (92–95), and to use this knowledge base to mount a 5-year community based participatory research prevention trial. The trial will enrol 239 youths, ages 12–18, in 5 rural remote Yup'ik communities in order to test effectiveness post intervention using a randomized dynamic wait list control design and to understand outcomes among subgroups (96).

A Danish study examined the health and social condition of adoptees in Greenland, where there are a large number of adoptees and children institutionalized (EoI # 1201). The study explored how adoption and collective care have an impact on well-being, family health and social conditions. Adoption is closely linked to social organization, identity, cultural openness and collective consciousness; this study identified settings in which adoption was linked to child neglect and lack of care. The study also examined parents’ and care givers’ control and coping strategies. The study concluded that contrary to findings related to adoptees in Western societies, being an adoptee in Greenland does not increase the risk for psychiatric admission (97).

### Health services delivery

The circumpolar regions experience unique challenges in the delivery of health services because of widely dispersed populations and geographic obstacles to service delivery. During IPY 2007–2008, opportunities were created for cross-border partnerships to explore needs related to service delivery. The Northern Forum, a forum of northern regional governments (www.northernforum.org), cooperated with the Alaska Federal Health Care Access Network (AFHCAN) to implement a strategic and innovative solution to address health care needs of 2 regions in the Arctic. Together the Northern Forum and AFHCAN facilitated cooperation in telemedicine technology expertise between Alaska, the Republic of Sakha and Khanty-Mansyisk region in Russia (EoI # 1270). The goal of the project was to promote the establishment of a mutually beneficial collaboration in telemedicine, telehealth, mobile medicine and distance learning in remote areas of the Russian north. This project is an important first step in both improving technologies to enhance access to care and utilize existing forums to promote cross border partnerships and activities.

Mental health services are also of importance in the north and efforts are required to enhance service delivery. The Northern Forum developed and promoted The Healthy Lifestyle Projects (EoI # 1271), which provided information exchange and training opportunities to advance care and treatment of Arctic residents with mental health issues. While the health service delivery research field is underdeveloped in the north, these projects identify key area of importance and play an important role as we begin to understand and develop best practices to improve services and programs in northern regions.

## Outreach education and communication

An important aspect of IPY was, and will continue to be, the promotion of education, outreach and communication, which will focus public and political attention on Arctic health issues; increase dialogue between researchers, policymakers and communities; increase distribution of scientific information to scientists and the public through conferences, symposia, workshops and a variety of electronic and printed media; and increase community involvement in research activities and foster a “new” generation of Arctic health scientists.

### Symposia and workshops

The IPY was highlighted by the occurrence of the 13th International Congress on Circumpolar Health held in Novosibirsk, Russian Federation, from 12–16 June 2006, the “Gateway to the IPY” for the circumpolar health community. This congress was put on by the International Union of Circumpolar Health (IUCH) and brought together 200 circumpolar health care professionals, workers, researchers, policymakers and indigenous community members. The meeting presented a forum for discussion on their respective visions and priorities for human health activities for the IPY and beyond. These discussions resulted in recommendations that emphasized the role of communities in research planning, research activities and the translation of research findings into actions that would benefit the health and well-being of Arctic communities (98).

The Women's Health Working Group of the IUCH was reactivated at that congress in June 2006 (EoI #1223). Participants identified at least 4 areas of mutual interest, including but not limited to (a) perinatal health systems and challenges (99,100); (b) infectious disease, particularly HPV and the new vaccine (35,36,101); (c) interpersonal violence prevention; and (d) health communication and health literacy (102). The Women's Health Working group maintains an active list of ~60 members to share resources and opportunities and sponsored a pre-congress seminar on Health Literacy and Northern Women's Health in Fairbanks in August 2012 that attracted ~50 participants. There have been several collaborative projects that have come out of this network, including a Pan-Arctic Inuit Wellness TV Series and an April 2012 special issue of the IJCH focused on Participatory Research and Ethics (103,104).

At the end of IPY, the 14th International Congress on Circumpolar Health was held in Yellowknife, Northwest Territories, Canada, from 12–16 July 2009. The Congress recognized the end of the Polar Year through its theme, “Securing the IPY legacy: from research to action”. While results from much of the research conducted over the IPY were still pending, the congress program contained a broad cross section of presenters, sessions and preliminary results from the IPY. The sessions allowed for complimentary perspectives of researchers, clinicians, community representatives and governments on numerous topics which impact public health, health services delivery, the research process and Indigenous wellness in our circumpolar regions. Presentations demonstrated instances where research findings are applied in numerous settings, with uptake by clinicians, community organizations and governments. Presentations also recognized the contributions of numerous stakeholders through the research process with a particular focus on community engagement and participatory methods (105).

The IPY Oslo Science conference (8–12 June 2010) was also a major venue for presenting all science conducted during the IPY 2007–8 (www.ipy-osc.no). Because this was the first time human health was a thematic area of research during an IPY, the meeting presented the opportunity to highlight human health activities conducted in both the Arctic and Antarctic during the IPY (www.ipy-osc.no/session/t4-1). At this conference, there were 6 human health sessions in all with a total of 27 presentations, including 31 poster presentations.

A follow-up IPY 2012 “From Knowledge to Action” Conference (22–27 April 2012) held in Montreal, Canada (www.ipy2012montreal.ca) and brought together over 2,000 Arctic and Antarctic researchers, policy- and decision-makers, and a broad range of other interested parties from academia, industry, non-government, education and circumpolar communities including indigenous peoples. The conference focused on the translation of IPY research findings to the development of an agenda for action for the future. The conference featured 6 human health and well-being sessions, a plenary panel on Communities and Health, and an action forum on Improving Access to Quality and Sustainable Health Care in Arctic Communities. Issues surrounding human health and well-being, food security, mitigation and adaptation will increasingly be the focus for science and public health work in the coming decades.

The Arctic like most other parts of the world has warmed substantially over the last few decades. The impacts of climate change on the health of Arctic residents will vary depending on such factors as age, socio-economic status, lifestyle, culture, location and capacity of the local health care infrastructures to adapt. It is likely that the most vulnerable will be those living close to the land in remote communities and those already facing health related challenges (106).

Climate change and health workshops (NI7) were convened in Anchorage, Alaska, as part of the 2008 Alaska Forum on the Environment (www.akforum.com) (107); a meeting in Moscow May 2008 organized by UNDP, WHP and UNEP resulted in report by the United Nations in the Russian Federation “Climate Change Impact on Public Health in the Russian Arctic” (www.unrussia.ru/en/documents) (108,109). A meeting coordinated by UNESCO and hosted by the Principality of Monaco in Monte Carlo, 3–6 March 2009, explored aspects of sustainable development in the Arctic in the face of climate change and called on the Arctic Council and the WHO to take action on human health recommendations identified by chapter 15 of the ACIA (106,110).

A joint AMAP and NCP symposium was held in Iqaluit, Nunavut, Canada, 10–12 June 2009 (FP # 145). At this meeting, the third NCP and AMAP Human Health Assessments reports on environmental contaminants were released, and the results were discussed (13,111,112). The symposium demonstrated that the overall management of contaminants issue in the Arctic globally through implementation of the Stockholm Convention and the United Nations Economic Commission for Europe Convention on Long-Range Trans-boundary Air Pollution Protocols has been effective in reducing the health risks to northern populations from environmental contaminants. While the results indicate that there are declines in many contaminants in several Arctic Regions, there are still indications that there may be subtle health effects (cardiovascular, immunological and behavioral) due to contaminants in some Arctic Indigenous populations. The symposium reemphasized the importance of biomonitoring of POPs and metals to track the efficiency of international treaties, biomonitoring of emerging contaminants, quality control of laboratory methods, health effects research, dietary choice, and risk perception and risk communication (113).

The Fogarty International Center at the National Institutes of Health (NIH) together with the US Arctic Research Commission (USARC) and other NIH institutes and CDC, organized a strategy setting conference on the “Behavioral and Mental Health Research in the Arctic” in Anchorage, AK, on 2–3 June 2009 (NI6). The purpose of this meeting was to develop a US Arctic Human Health Research Strategy that will advise the Interagency Arctic Research Policy Committee (IARPC) on the development of an Arctic Human Health Research Plan. This meeting engaged Arctic health stakeholders including US government, scientific and tribal community leaders and international scientists in behavioral and mental health with discussions of current knowledge and gaps in research, with a particular focus on improving our understanding of the risk factors for and barriers to reduce suicide and other behavioral and mental health ailments among Arctic populations. The conference outcome will be a strategy plan that will include specific goals and methods, as well as discussion of potential future research and research training activities on behavioral and mental health in the Arctic (84,85).

## Electronic and print media

### Dissemination in scientific community

While the activities of the polar years focused on study implementation and data collection, analysis and dissemination of findings will be ongoing for years to come. During the IPY a number of summary and synthesis documents were created. The International Journal of Circumpolar Health (www.circumpolarhealthjournal.net) produced a series of Circumpolar Health Supplements on topics of general interest and related to the IPY themes (EoI # 1046). To date, 7 supplements have been published as contributions to the IPY: (a) Anthropology and Health of Indigenous Peoples of Northern Russia (114); (b) Diet and Contaminants in Greenland (115); (c) Circumpolar Health Indicators (116); (d) International Circumpolar Surveillance: Prevention and Control of Infectious Diseases (7); (e) Behavioral and Mental Health Research in the Arctic: Strategy Setting Meeting (84); (f) The Arctic Human Health Initiative (117), and (g) the Proceedings of the 14th International Congress on Circumpolar Health (105).

The IPY activities related to human health primarily focused on Arctic regions with permanent human inhabitants. However, some health research is conducted in Antarctic regions using transient populations largely comprising of scientists, explorers and occupational workers. The human health needs in these populations tend to focus on emergency medicine, telehealth, rescue and expedition medicine and human response to isolation, cold and remote environments. Populations are small, so studies tend to be descriptive or qualitative. Despite the high level of scientific activity in these regions, scientific programs that explore the human health of these populations were underdeveloped during the IPY. In an attempt to capture health research conducted at both Poles, a Special IPY issue of Rural and Remote Health (www.rrh.org.au) *Human Health at the Ends of the Earth* was published in 2010 (118).

The International Network for Circumpolar Health Research produced a book *Health Transitions in Arctic Populations* (22) with contributions from 23 scientists and health care practitioners from all the Arctic countries. It synthesized existing knowledge on the health status of all the circumpolar regions and populations, with specific focus on the indigenous Sámi, Dene and Inuit people, their determinants, and strategies for improving their health.

In 2011, the IPY Joint Committee published a summary volume on the context, motivations, innovations, planning, implementation and outcomes of all activities conducted during the IPY 2007–2009. An estimated 50,000 researchers, local observers, educator's students and support personnel from more than 60 nations were involved in the 228 IPY projects and related national initiatives. Human Health activities are summarized in Chapter 2.11 (119), and are updated in this report.

### Multimedia and knowledge sharing

The AHHI facilitated the development of the Arctic Health website www.arctichealth.org as a central source for information on diverse aspects of the Arctic environment and the health of northern peoples. The site gives access to health information from hundreds of local, state, national and international agencies, as well as from professional societies and universities. In addition, the Arctic Health Publications Database (currently more than 96,000 records) provides access to Arctic-specific articles, out of print publications and information from special collections held in the Alaska Medical Library.

During IPY, a concept for a circumpolar health portal, was developed (www.circumpolarhealth.org). This project is exploring the feasibility of a coordinated venue to capture and promote the activities of circumpolar health organizations and initiatives. The website also incorporates Facebook and Twitter and has dedicated channels for You Tube, podcasts and Flickr. These mechanisms allow for storage and access of photos, audio files and video. These tools are especially valuable to share information and outputs related to youth driven and participatory research projects.

In addition to web-based media, radio and TV still play an important role in the sharing of information with circumpolar residents (NI10). A series of 3 live TV call-in shows on Inuit wellness was developed under the umbrella of the Pan-Arctic Interactive Communications Health Project. TV programs were produced and focused on the current health issues of importance to Inuit, including (a) Inuit men's health and wellness; (b) Inuit maternal care; and (c) Inuit youth and coping. Each show was moderated and featured panel discussions about programs and research with community representatives and physicians, video vignettes and interactions with the studio audience and participants by Skype, phone and e-mail. The television broadcasts reached a wide audience by airing on networks in Canada and Alaska. This project was an innovative, multidimensional, collaborative health communication project that raised both interest and awareness about complex health conditions in the North and stimulated community dialogue and potential for both local and regional collaborative action. Ongoing evidence-based resources for health education and community action developed through this program were assembled and archived in digital format (www.naho.ca/inuit/e/TVseries) to increase accessibility for otherwise isolated individuals and remote communities.

## Education and training initiatives

Education and training in the discipline of circumpolar health is as varied and broad as the number of topics related to human health which are explored in circumpolar regions. Thus, education and training activities through the polar years have tended to be cross-cutting and integrated in research programs. Activities have included the support of graduate students and training of community partners. Many health research initiatives now employ community based participatory methods in which training in research methods, data collection and dissemination practices are integral components of the methodology. Examples of community participation have been demonstrated in programs such as the Inuit Health Survey (NI2), Healthy Foods North project (NI9) and the Inuit Cohort (NI8), an education initiative to promote graduate education for Inuit. All these initiatives are important as research methods are improved to incorporate academic and community perspectives. The evaluation of the Pan-Arctic Inuit Wellness TV Series project provides specific lessons to build a strong foundation for a community-professional-academic partnership (120).

In addition, the Centre for Arctic Medicine, Thule Institute, University of Oulu, Finland (http://arctichealth.oulu.fi), has a research and education program dedicated to circumpolar health and well-being, the main focus of the research projects are environmental health, marginalization and mental health (EoI# 1045). It is delivered in close collaboration with the University of the Arctic (www.uarctic.org). The program offers both PhD and Master's programs in the field of health and well-being in the circumpolar regions. The International Master's program started in 2008 with 14 students and the third set of 15 students starts on September. Three graduated students continue their studies as PhD students. Other partners of the Master's program that provided courses towards the degree program include the Center for Health Education (Nuuk, Greenland), Luleå University of Technology (Luleå, Sweden), Northern Medical State University (Arkhangeslsk, Russia), NARFU (Arkhangelsk, Russia), University of Lapland (Rovaniemi, Finland), and University of Manitoba (Winnipeg, Canada), as well as the Cross Border University of Barents area. The University of the Arctic's international PhD program “Arctic Health and Well-being” started in 2012. The main work for course development continues through 2013 and institutional accreditations will be sought, while in 2014 the student recruitments will be completed. The Centre for Arctic Medicine is arranging with partners for summer and winter courses for PhD students, first at the University of Alaska Anchorage MPH program, and then at the Summer Institute in Circumpolar Health Research in Copenhagen in May 2010 (http://sichr.circumpolarhealth.org), continuing in Oulu, November 2010, in Kautokeino, March 2011, in Oulu, June 2011, in Abisko Research Station, April 2012, and in Oulu, November 2012, and the next will be in Abisko Research Station May 2013 and Nuuk, Greenland, September 2013.

## Securing the legacy of IPY 2007–2009

The overall goal of the AHHI was to increase awareness and visibility of human health concerns of Arctic peoples, foster human health research and promote health strategies that will improve health and well-being of all Arctic residents. The AHHI proved to be an effective exercise in identifying and featuring health research activities during IPY for raising the profile or Arctic human health within national governments and has highlighted the need within the Arctic Council for an ongoing emphasis, action and strategic direction for addressing critical areas of human health in the Arctic. This need was recognized during the Norwegian Arctic Council Chairmanship (2006–2009) and resulted in the formation in 2010 of the Arctic Human Health Expert Group (AHHEG), with professional circumpolar expertise in the areas of health systems, services and policy, social cultural and economic aspects of health, indigenous and traditional knowledge, physical and social science including behavioral and mental health and human biology, and environmental health, contaminants, and climate change (121).

The AHHEG will assist the Arctic Council in better coordinating its human health activities, by:

- Identifying priority projects that will result in improved health;
- Engaging the appropriate subject matter experts to evaluate potential actions and collaborate on priority projects;
- Monitoring project progress; and
- Improving the Arctic Councils’ ability to translate knowledge gained into meaningful actions that will benefit communities, and that will result in health improvement.

Early priorities already identified by the AHHEG include:

- Behavioral and mental health including youth suicides;
- Diet and nutrition with an emphasis on food security, safe water, obesity diabetes and cardiovascular diseases;
- Health care in indigenous populations, including culturally appropriate care of the elderly;
- Inequalities in health; and
- The human health impacts of climate change.

The importance of human health in the Arctic was again recognized on 16 February 2011, when the Government of Greenland, at the end of the Greenland/Danish Arctic Council Chairmanship, hosted the first Arctic Human Health Ministerial meeting in Nuuk, Greenland. This meeting resulted in the Arctic Health Declaration, a document signed by health ministerial representatives of the governments of Canada, Denmark, Greenland, Iceland, Norway, Sweden, the Russian Federation, the USA and the Faroe Islands (122). This declaration is intended to guide international cooperation on and national priorities for Arctic human health research and health promotion activities for many years to come.

Human health is now a critical component of the Arctic Council's sustainable development program. The AHHEG within the SDWG will continue to explore ways to ensure greater integration of human health activities, strengthen cooperation and collaborations between Arctic Council working groups and other Arctic cooperatives, and promote the translation of research into actions that will improve the health of all Arctic residents (123).
